# Draft Genome Sequence of *Bacillus megaterium* Type Strain ATCC 14581

**DOI:** 10.1128/genomeA.01124-14

**Published:** 2014-11-13

**Authors:** Gitanjali Arya, Nicholas Petronella, Jennifer Crosthwait, Catherine D. Carrillo, Philip S. Shwed

**Affiliations:** a Environmental Health Science and Research Bureau, Environmental and Radiation Health Sciences Directorate, HECSB, Health Canada, Ottawa, Ontario, Canada; b Bureau of Food Surveillance and Science Integration, Food Directorate, Health Canada, Ottawa, Ontario, Canada; c Canadian Food Inspection Agency, Ottawa, Ontario, Canada

## Abstract

*Bacillus megaterium* is a Gram-positive, rod-shaped, spore-forming bacterium of biotechnological importance. Here, we report a 5.7-Mbp draft genome sequence of *B. megaterium* ATCC 14581, which is the type strain of the species.

## GENOME ANNOUNCEMENT

*Bacillus megaterium* is a ubiquitous environmental bacterium. It is economically important for the production of recombinant proteins and vitamins, as well as for bioremediation activities (1). The microbe was first described by Anton De Bary in 1884 (2), and later the species was defined in detail by Ford and Lawrence (3) in 1916, from an environmental source isolate. De Bary’s (2) culture of the species went missing and was presumed lost (4, 5); thus, Ford’s strain 19 supplied to contemporary culture collections became the type strain. *B. megaterium* ATCC 14581^T^ (also DSM32^T^, NCIMB 9376, NCTC 10342^T^, BCRC 10608^T^) features typical traits of the species, and its genome sequence, reported here, may serve as a reference for comparative genomic analysis.

Whole-genome sequencing of *B. megaterium* ATCC 14581^T^ was performed on the Illumina HiSeq platform at the Michael Smith Genome Sciences Centre (Vancouver, Canada). A total of 2,908,022 paired-end reads (2 × 100 bp) were generated and assembled *de novo* using Velvet 1.2.10 (6) and VelvetOptimiser 2.2.5 (http://bioinformatics.net.au/software.velvetoptimiser.shtml). Various hash lengths between 31 and 91 were tested, and the optimal k-mer size was found to be 71 bp, resulting in a total of 122 contigs, with an *N*_50_ length of 324,443 bp and an average 50× genome coverage. After eliminating the contigs sized ≤199 nucleotides (nt) (http://www.ncbi.nlm.nih.gov/genbank/genomesubmit), the draft genome sequence contained 78 contigs. The draft whole-genome size (WGS) is 5.7 Mbp, with a G+C content of 37%, and the assembly appears to be larger than other finished genomes of *B. megaterium*. The draft genome sequence was annotated using the RAST server (7), which predicted 5,955 coding sequences (CDSs) and 84 RNA genes, and it mapped genes to 486 subsystems.

Consistent with the previously published genomes of *B. megaterium*, that of ATCC 14581^T^ possesses a large syntenic region around the origin of replication, a characteristic feature observed in the genome architecture of the sporulating *Bacillus* species (8, 9). The draft genome of ATCC 14581^T^ includes genes encoding heavy metal and antibiotic resistance, as well as iron acquisition systems, which are known to be associated with the adaptation and survival of *B. megaterium* under diverse environmental conditions (10, 11). The draft genome is predicted to contain 544 CDSs in the subsystem of carbohydrate utilization, consisting of metabolic pathways, including glyoxylate cycle, glycolysis, and the tricarboxylic acid cycle. The ATCC 14581^T^ CDSs were mapped to a number of transmembrane transport systems, including 18 CDSs involved in ATP-binding cassette transporters, 15 CDSs involved in cation transport (copper, magnesium, and nickel), and 34 CDSs involved in protein translocation. Compared to the current representative genome for the species, strain DSM 319, some of the unique genes found in ATCC 14581^T^ include mobile genetic elements (prophages and transposases) and proteins involved in phosphotransferase uptake systems for the catabolism of the polyalcohol galactitol and β-glucoside sugar substrates, indicating the ability of *B. megaterium* to utilize a wide variety of carbon sources. The draft genome of ATCC 14581^T^ will facilitate future biotechnology applications and comparative genomic and phylogenetic analyses involving strains from different origins.

### Nucleotide sequence accession numbers.

The whole-genome shotgun project of *B. megaterium* ATCC 14581^T^ has been deposited at DDBJ/EMBL/GenBank under the accession no. JJMH00000000. The version described in the paper is JJMH01000000.
